# 2-(2-Hy­droxy­phen­yl)-1,3-benzothia­zole-6-carbaldehyde

**DOI:** 10.1107/S1600536811040712

**Published:** 2011-10-08

**Authors:** Kew-Yu Chen, Tzu-Chien Fang, Ming-Jen Chang, Hsing-Yang Tsai, Ming-Hui Luo

**Affiliations:** a Department of Chemical Engineering, Feng Chia University, 40724 Taichung, Taiwan

## Abstract

The mol­ecule of the title compound, C_14_H_9_NO_2_S, is nearly planar, the maximum atomic deviation being 0.081 (2) Å. An intra­molecular O—H⋯N bond generates an *S*(6) ring motif. In the crystal, inversion-related mol­ecules linked by a pair of weak C—H⋯O hydrogen bonds form a supra­molecular dimer. π–π stacking is observed between the thia­zole and benzene rings of adjacent mol­ecules, the centroid–centroid distance being 3.7679 (9) Å.

## Related literature

For the spectroscopy and preparation of the title compound, see: Hsieh *et al.* (2008 ▶). For the spectroscopy and applications of benzoxazole and benzothia­zole derivatives, see: Chen & Pang (2009 ▶, 2010 ▶); Hrobáriková *et al.* (2010 ▶); Kim *et al.* (2010*a* ▶,*b* ▶); Lijima *et al.* (2010 ▶); Lim *et al.* (2011 ▶); López-Ruiz *et al.* (2011 ▶); Tanaka *et al.* (2001 ▶). For related structures, see: Tong (2005 ▶); Hahn *et al.* (1998 ▶). For graph-set theory, see: Bernstein *et al.* (1995 ▶).
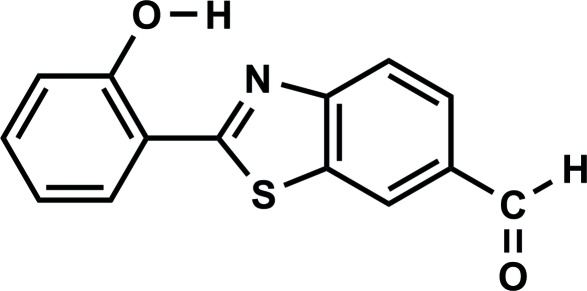


## Experimental

### 

#### Crystal data

C_14_H_9_NO_2_S
*M* *_r_* = 255.28
Monoclinic, 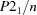

*a* = 8.2645 (3) Å
*b* = 5.6449 (2) Å
*c* = 23.8341 (9) Å
β = 98.147 (2)°
*V* = 1100.69 (7) Å^3^
*Z* = 4
Mo *K*α radiation
μ = 0.29 mm^−1^
*T* = 150 K
0.38 × 0.14 × 0.04 mm

#### Data collection

Bruker SMART CCD area-detector diffractometer
Absorption correction: multi-scan (*SADABS*; Bruker, 2001 ▶) *T*_min_ = 0.882, *T*_max_ = 0.992
8427 measured reflections
1943 independent reflections
1333 reflections with *I* > 2σ(*I*)
*R*_int_ = 0.042

#### Refinement

*R*[*F* ^2^ > 2σ(*F* ^2^)] = 0.029
*wR*(*F* ^2^) = 0.058
*S* = 0.90
1943 reflections
168 parameters
1 restraint
H atoms treated by a mixture of independent and constrained refinement
Δρ_max_ = 0.22 e Å^−3^
Δρ_min_ = −0.27 e Å^−3^

### 

Data collection: *SMART* (Bruker, 2001 ▶); cell refinement: *SAINT* (Bruker, 2001 ▶); data reduction: *SAINT*; program(s) used to solve structure: *SHELXS97* (Sheldrick, 2008 ▶); program(s) used to refine structure: *SHELXL97* (Sheldrick, 2008 ▶); molecular graphics: *ORTEP-3 for Windows* (Farrugia, 1997 ▶); software used to prepare material for publication: *WinGX* (Farrugia, 1999 ▶).

## Supplementary Material

Crystal structure: contains datablock(s) I, global. DOI: 10.1107/S1600536811040712/xu5345sup1.cif

Structure factors: contains datablock(s) I. DOI: 10.1107/S1600536811040712/xu5345Isup2.hkl

Supplementary material file. DOI: 10.1107/S1600536811040712/xu5345Isup3.cml

Additional supplementary materials:  crystallographic information; 3D view; checkCIF report

## Figures and Tables

**Table 1:** Hydrogen-bond geometry (Å, °)

*D*—H⋯*A*	*D*—H	H⋯*A*	*D*⋯*A*	*D*—H⋯*A*
O2—H2⋯N1	0.89 (2)	1.81 (2)	2.6228 (18)	150 (2)
C5—H5⋯O2^i^	0.93	2.61	3.293 (2)	130

Symmetry code: (i) 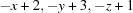.

**Fig. 1.**
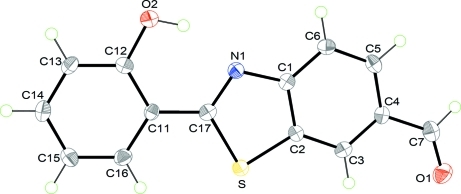
The molecular structure of the title compound, showing 50% probability displacement ellipsoids.

**Fig. 2.**
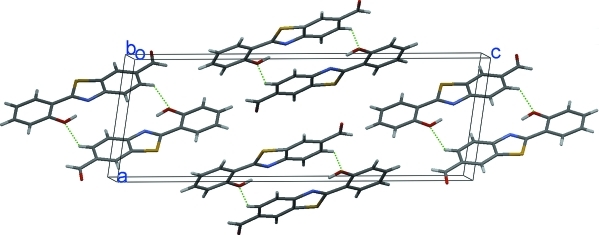
A section of the crystal packing of the title compound, viewed down the b axis. Green dashed lines denote the intermolecular C—H···O hydrogen bonds.

## supplementary crystallographic information

### Comment

The excited-state intramolecular proton transfer (ESIPT) reaction of 2-(2-hydroxyphenyl)benzoxazole and 2-(2-hydroxyphenyl)benzothiazole derivatives has been investigated for past years (Hsieh *et al.*, 2008; Kim *et al.*, 2010*a*,*b*; Lijima *et al.*, 2010; López-Ruiz *et al.*, 2011), which incorporates transfer of a hydroxy proton to the imine nitrogen through a intramolecular six-membered-ring hydrogen-bonding system (Chen *et al.*, 2009, 2010). The unusual photophysical property of the resulting proton-transfer tautomer has found many important applications (Hrobáriková *et al.*, 2010; Lim *et al.*, 2011; Tanaka *et al.*, 2001).

The molecular structure of the title compound (HBT) is shown in Figure 1. The molecule is nearly planar, which is consistent with previous studies (Tong, 2005; Hahn *et al.*, 1998). HBT possesses an intramolecular O—H···N hydrogen bond (Table 1), which generates an S(6) ring motif (Bernstein *et al.*, 1995). In the crystal (Figure 2), inversion-related molecules are linked by a pair of weak C—H···O hydrogen bonds, forming a cyclic dimers with *R*_2_^2^(18) graph-set motif. π-π stacing is observed between thiazole and C1^i^-benzene rings of adjacent molecules [symmetry code: (i): 2-x,2-y,1-z], the centroid-to-centroid distance being 3.7679 (9) Å.

### Experimental

The title compound was synthesized according to the literature (Hsieh *et al.*, 2008). Yellow needle-shaped crystals suitable for the crystallographic studies reported here were isolated over a period of five weeks by slow evaporation from the chloroform solution.

### Refinement

H atoms bonded to O and C atoms were located in a difference electron density map. The hydroxy H atom and the C_sp3_ H atoms were freely refined, and the C_sp2_ H atoms repositioned geometrically and refined using a riding model, [C—H = 0.93 Å and *U*_iso_(H) = 1.2*U*_eq_(C)].

### Crystal data

**Table:**

C_14_H_9_NO_2_S	*F*(000) = 528
*M**_r_* = 255.28	*D*_x_ = 1.541 Mg m^−^^3^
Monoclinic, *P*2_1_/*n*	Mo *K*α radiation, λ = 0.71073 Å
Hall symbol: -p 2yn	Cell parameters from 2297 reflections
*a* = 8.2645 (3) Å	θ = 2.5–25.7°
*b* = 5.6449 (2) Å	µ = 0.29 mm^−^^1^
*c* = 23.8341 (9) Å	*T* = 150 K
β = 98.147 (2)°	Plate, yellow
*V* = 1100.69 (7) Å^3^	0.38 × 0.14 × 0.04 mm
*Z* = 4	

### Data collection

**Table:**

Bruker SMART CCD area-detector diffractometer	1943 independent reflections
Radiation source: fine-focus sealed tube	1333 reflections with *I* > 2σ(*I*)
graphite	*R*_int_ = 0.042
φ and ω scans	θ_max_ = 25.0°, θ_min_ = 1.7°
Absorption correction: multi-scan (*SADABS*; Bruker, 2001)	*h* = −9→9
*T*_min_ = 0.882, *T*_max_ = 0.992	*k* = −6→3
8427 measured reflections	*l* = −28→28

### Refinement

**Table:**

Refinement on *F*^2^	Secondary atom site location: difference Fourier map
Least-squares matrix: full	Hydrogen site location: inferred from neighbouring sites
*R*[*F*^2^ > 2σ(*F*^2^)] = 0.029	H atoms treated by a mixture of independent and constrained refinement
*wR*(*F*^2^) = 0.058	*w* = 1/[σ^2^(*F*_o_^2^) + (0.0251*P*)^2^] where *P* = (*F*_o_^2^ + 2*F*_c_^2^)/3
*S* = 0.90	(Δ/σ)_max_ < 0.001
1943 reflections	Δρ_max_ = 0.22 e Å^−^^3^
168 parameters	Δρ_min_ = −0.27 e Å^−^^3^
1 restraint	Extinction correction: *SHELXL97* (Sheldrick, 2008), Fc^*^=kFc[1+0.001xFc^2^λ^3^/sin(2θ)]^-1/4^
Primary atom site location: structure-invariant direct methods	Extinction coefficient: 0.0014 (2)

### Special details

**Table:**

Geometry. All e.s.d.'s (except the e.s.d. in the dihedral angle between two l.s. planes) are estimated using the full covariance matrix. The cell e.s.d.'s are taken into account individually in the estimation of e.s.d.'s in distances, angles and torsion angles; correlations between e.s.d.'s in cell parameters are only used when they are defined by crystal symmetry. An approximate (isotropic) treatment of cell e.s.d.'s is used for estimating e.s.d.'s involving l.s. planes.
Refinement. Refinement of *F*^2^ against ALL reflections. The weighted *R*-factor *wR* and goodness of fit *S* are based on *F*^2^, conventional *R*-factors *R* are based on *F*, with *F* set to zero for negative *F*^2^. The threshold expression of *F*^2^ > σ(*F*^2^) is used only for calculating *R*-factors(gt) *etc*. and is not relevant to the choice of reflections for refinement. *R*-factors based on *F*^2^ are statistically about twice as large as those based on *F*, and *R*- factors based on ALL data will be even larger.

### Fractional atomic coordinates and isotropic or equivalent isotropic displacement parameters (Å^2^)

**Table:**

	*x*	*y*	*z*	*U*_iso_*/*U*_eq_	
S	0.74344 (6)	0.79801 (8)	0.392532 (18)	0.02213 (16)	
O1	0.51794 (15)	0.6205 (2)	0.59718 (5)	0.0317 (4)	
O2	1.05490 (15)	1.4470 (2)	0.35839 (5)	0.0272 (3)	
N1	0.88683 (16)	1.1973 (2)	0.42282 (6)	0.0183 (3)	
C1	0.81872 (19)	1.1165 (3)	0.46928 (7)	0.0167 (4)	
C2	0.7362 (2)	0.8986 (3)	0.46067 (7)	0.0169 (4)	
C3	0.66326 (19)	0.7940 (3)	0.50315 (7)	0.0189 (4)	
H3	0.6076	0.6510	0.4970	0.023*	
C4	0.67509 (19)	0.9071 (3)	0.55528 (7)	0.0183 (4)	
C5	0.7578 (2)	1.1247 (3)	0.56406 (7)	0.0211 (4)	
H5	0.7644	1.1987	0.5992	0.025*	
C6	0.82896 (19)	1.2299 (3)	0.52162 (7)	0.0204 (4)	
H6	0.8830	1.3741	0.5277	0.024*	
C7	0.5997 (2)	0.7988 (3)	0.60128 (7)	0.0252 (4)	
H7	0.6162	0.8736	0.6364	0.030*	
C11	0.91907 (19)	1.0821 (3)	0.32631 (7)	0.0168 (4)	
C12	1.0157 (2)	1.2802 (3)	0.31764 (7)	0.0190 (4)	
C13	1.07588 (19)	1.3083 (3)	0.26659 (7)	0.0219 (4)	
H13	1.1400	1.4394	0.2611	0.026*	
C14	1.04146 (19)	1.1439 (3)	0.22413 (7)	0.0229 (5)	
H14	1.0826	1.1644	0.1901	0.027*	
C15	0.9459 (2)	0.9477 (3)	0.23149 (7)	0.0239 (5)	
H15	0.9225	0.8371	0.2026	0.029*	
C16	0.8862 (2)	0.9183 (3)	0.28209 (7)	0.0222 (4)	
H16	0.8223	0.7863	0.2870	0.027*	
C17	0.85778 (19)	1.0478 (3)	0.38021 (7)	0.0180 (4)	
H2	1.005 (2)	1.407 (4)	0.3877 (7)	0.071 (8)*	

### Atomic displacement parameters (Å^2^)

**Table:**

	*U*^11^	*U*^22^	*U*^33^	*U*^12^	*U*^13^	*U*^23^
S	0.0278 (3)	0.0195 (3)	0.0199 (3)	−0.0044 (2)	0.0062 (2)	−0.0013 (2)
O1	0.0349 (8)	0.0318 (8)	0.0295 (8)	−0.0074 (7)	0.0085 (7)	0.0053 (6)
O2	0.0346 (8)	0.0245 (8)	0.0233 (8)	−0.0092 (6)	0.0070 (7)	−0.0026 (6)
N1	0.0198 (9)	0.0164 (8)	0.0186 (8)	0.0005 (7)	0.0025 (7)	0.0009 (7)
C1	0.0154 (10)	0.0160 (10)	0.0181 (10)	0.0040 (8)	0.0003 (8)	0.0037 (8)
C2	0.0172 (10)	0.0169 (10)	0.0165 (10)	0.0029 (8)	0.0016 (8)	0.0006 (8)
C3	0.0162 (10)	0.0168 (10)	0.0233 (11)	0.0011 (8)	0.0016 (8)	0.0021 (9)
C4	0.0170 (10)	0.0194 (10)	0.0187 (10)	0.0043 (8)	0.0034 (8)	0.0037 (8)
C5	0.0226 (11)	0.0225 (11)	0.0179 (11)	0.0051 (9)	0.0019 (9)	−0.0020 (8)
C6	0.0209 (11)	0.0178 (10)	0.0220 (11)	−0.0004 (8)	0.0011 (8)	−0.0001 (8)
C7	0.0241 (12)	0.0299 (11)	0.0215 (11)	0.0069 (10)	0.0030 (9)	0.0008 (9)
C11	0.0162 (10)	0.0173 (10)	0.0171 (10)	0.0008 (8)	0.0028 (8)	0.0023 (8)
C12	0.0183 (10)	0.0184 (10)	0.0194 (10)	0.0019 (9)	−0.0003 (8)	−0.0006 (9)
C13	0.0207 (11)	0.0220 (10)	0.0233 (11)	−0.0037 (8)	0.0048 (9)	0.0048 (9)
C14	0.0202 (11)	0.0292 (12)	0.0201 (11)	0.0032 (9)	0.0060 (9)	0.0051 (9)
C15	0.0279 (12)	0.0240 (11)	0.0198 (11)	0.0016 (9)	0.0040 (9)	−0.0030 (8)
C16	0.0238 (11)	0.0184 (10)	0.0244 (11)	−0.0023 (9)	0.0038 (9)	0.0011 (9)
C17	0.0146 (10)	0.0163 (10)	0.0228 (11)	0.0030 (8)	0.0016 (8)	0.0022 (8)

### Geometric parameters (Å, °)

**Table:**

S—C2	1.7296 (16)	C5—H5	0.9300
S—C17	1.7454 (17)	C6—H6	0.9300
O1—C7	1.2087 (19)	C7—H7	0.9300
O2—C12	1.3588 (19)	C11—C16	1.400 (2)
O2—H2	0.890 (14)	C11—C12	1.406 (2)
N1—C17	1.3160 (18)	C11—C17	1.459 (2)
N1—C1	1.3882 (19)	C12—C13	1.387 (2)
C1—C6	1.394 (2)	C13—C14	1.373 (2)
C1—C2	1.407 (2)	C13—H13	0.9300
C2—C3	1.382 (2)	C14—C15	1.386 (2)
C3—C4	1.388 (2)	C14—H14	0.9300
C3—H3	0.9300	C15—C16	1.376 (2)
C4—C5	1.407 (2)	C15—H15	0.9300
C4—C7	1.469 (2)	C16—H16	0.9300
C5—C6	1.375 (2)		
			
C2—S—C17	89.10 (8)	C4—C7—H7	117.4
C12—O2—H2	107.2 (14)	C16—C11—C12	117.93 (15)
C17—N1—C1	110.79 (14)	C16—C11—C17	121.39 (15)
N1—C1—C6	125.67 (16)	C12—C11—C17	120.67 (15)
N1—C1—C2	114.43 (15)	O2—C12—C13	117.94 (15)
C6—C1—C2	119.88 (16)	O2—C12—C11	121.94 (15)
C3—C2—C1	121.30 (16)	C13—C12—C11	120.11 (16)
C3—C2—S	128.62 (14)	C14—C13—C12	120.42 (16)
C1—C2—S	110.07 (12)	C14—C13—H13	119.8
C2—C3—C4	118.44 (16)	C12—C13—H13	119.8
C2—C3—H3	120.8	C13—C14—C15	120.61 (16)
C4—C3—H3	120.8	C13—C14—H14	119.7
C3—C4—C5	120.40 (16)	C15—C14—H14	119.7
C3—C4—C7	119.55 (16)	C16—C15—C14	119.28 (16)
C5—C4—C7	120.05 (16)	C16—C15—H15	120.4
C6—C5—C4	121.13 (16)	C14—C15—H15	120.4
C6—C5—H5	119.4	C15—C16—C11	121.64 (16)
C4—C5—H5	119.4	C15—C16—H16	119.2
C5—C6—C1	118.84 (16)	C11—C16—H16	119.2
C5—C6—H6	120.6	N1—C17—C11	123.12 (15)
C1—C6—H6	120.6	N1—C17—S	115.59 (12)
O1—C7—C4	125.29 (17)	C11—C17—S	121.27 (12)
O1—C7—H7	117.4		

### Hydrogen-bond geometry (Å, °)

**Table:**

*D*—H···*A*	*D*—H	H···*A*	*D*···*A*	*D*—H···*A*
O2—H2···N1	0.89 (2)	1.81 (2)	2.6228 (18)	150 (2)
C5—H5···O2^i^	0.93	2.61	3.293 (2)	130

Symmetry codes:  (i) −*x*+2, −*y*+3, −*z*+1.
